# Exploring New Potential Anticancer Activities of the G-Quadruplexes Formed by [(GTG_2_T(G_3_T)_3_] and Its Derivatives with an Abasic Site Replacing Single Thymidine

**DOI:** 10.3390/ijms22137040

**Published:** 2021-06-30

**Authors:** Antonella Virgilio, Daniela Benigno, Annalisa Pecoraro, Annapina Russo, Giulia Russo, Veronica Esposito, Aldo Galeone

**Affiliations:** Department of Pharmacy, University of Naples Federico II, 80131 Napoli, Italy; antonella.virgilio@unina.it (A.V.); daniela.benigno@unina.it (D.B.); annalisa.pecoraro@unina.it (A.P.); annapina.russo@unina.it (A.R.); giulia.russo@unina.it (G.R.); galeone@unina.it (A.G.)

**Keywords:** G-quadruplex, abasic site, aptamers, antiproliferative activity

## Abstract

In this paper, we report our investigations on five T30175 analogues, prepared by replacing sequence thymidines with abasic sites (S) one at a time, in comparison to their natural counterpart in order to evaluate their antiproliferative potential and the involvement of the residues not belonging to the central core of stacked guanosines in biological activity. The collected NMR (Nuclear Magnetic Resonance), CD (Circular Dichroism), and PAGE (Polyacrylamide Gel Electrophoresis) data strongly suggest that all of them adopt G-quadruplex (G4) structures strictly similar to that of the parent aptamer with the ability to fold into a dimeric structure composed of two identical G-quadruplexes, each characterized by parallel strands, three all-*anti-*G-tetrads and four one-thymidine loops (one bulge and three propeller loops). Furthermore, their antiproliferative (MTT assay) and anti-motility (wound healing assay) properties against lung and colorectal cancer cells were tested. Although all of the oligodeoxynucleotides (ODNs) investigated here exhibited anti-proliferative activity, the unmodified T30175 aptamer showed the greatest effect on cell growth, suggesting that both its characteristic folding in dimeric form and its presence in the sequence of all thymidines are crucial elements for antiproliferative activity. This straightforward approach is suitable for understanding the critical requirements of the G-quadruplex structures that affect antiproliferative potential and suggests its application as a starting point to facilitate the reasonable development of G-quadruplexes with improved anticancer properties.

## 1. Introduction

**Figure 1:**
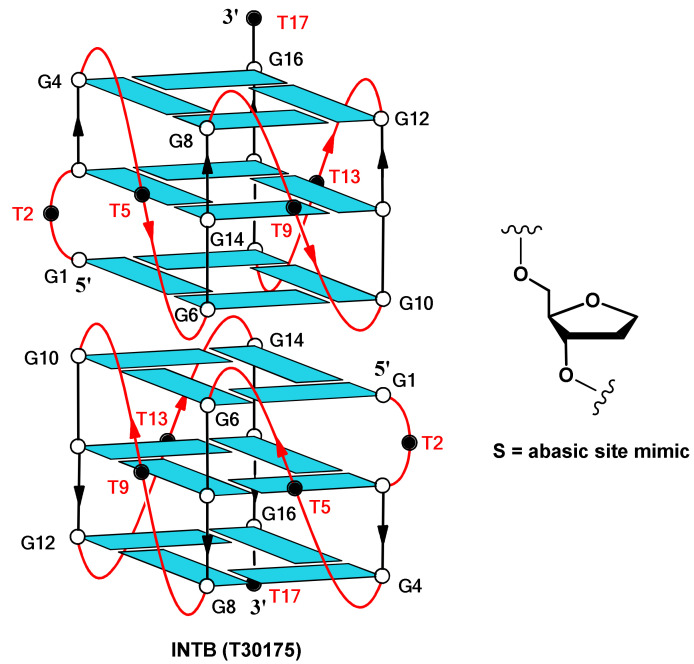
Schematic representation of the parallel-stranded G-quadruplex dimer structure of T30175 (**INT-B**) and the chemical structure of the abasic site mimic (S), introduced in positions 2, 5, 9, 13, and 17. All guanosines adopt anti glycosidic conformations (in light blue). The thymidines are represented as black circles and are labeled in red.

DNA and RNA aptamers are small oligonucleotide ligands that bind their target, such as small molecules, proteins, nucleic acids, and even cells, tissues, and organisms, with remarkable affinity and specificity. The in vitro selection technique named SELEX (Systematic Evolution of Ligands by Exponential Enrichment), a combinatorial method to select aptamers, revealed several highly specific G-quadruplexes forming oligonucleotide aptamers (G4-aptamers) against a variety of protein targets [1,2]. This is not particularly surprising, taking into account that G4 structures are very stable nucleic acid secondary conformations [3]. The folding in G4 conformations requires G-rich sequences that can form quadrangular planar arrangements of four guanosines (known as G-tetrads) connected through eight H-bonds overall. The stacking of two or more G-tetrads and the coordination of a metal cation in the central channel further contribute to stabilizing the structure. G-quadruplexes occur naturally in telomers, as well as in promoter regions. Many regulators of transcription, translation, replication, signal transduction, recombination, and other key biological processes in cells are cellular proteins that specifically interact with these non-canonical structures [4,5,6]. Consequently, exogenous G4 aptamers can affect the activity of G4-recognizing proteins, constituting one of the most investigated classes of protein ligands of considerable relevance in research areas such as diagnostics, therapeutics, biosensing, or gene silencing. The three-dimensional structure of aptamers is the base of their high specificity, together with the peculiar arrangement of loops and stems and the hydrogen bonds that stabilize the structure. It is noteworthy that the presence of one- or two-residue loops linking the G-runs and protruding outwardly is characteristic of most of G4 aptamers whose structure has been ascertained or hypothesized, such as the thrombin-targeting anticoagulant aptamers TBA (Thrombin Binding Aptamer) and NU172 [7,8], the anti-HIV integrase aptamers T30923 [G_3_(TG_3_)_3_T] [9] and T30175 [GTG_2_(TG_3_)_3_T] [10], and the nucleolin-targeting antiproliferative aptamer AS1411 [11]. Keeping in mind their topologies [12], it is very plausible that most of the structural stability of these G4 aptamers is due to the compact core formed by the stacked G-tetrads, while the external more accessible loop residues are mostly involved in the interaction with the target protein. Concerning the TBA/thrombin interaction, a significant number of investigations, based on many chemical modifications designed in order to improve biological properties, thermal stability, and resistance in biological environments of TBA [13], have pointed out that the regions of the aptamer mainly implicated in the interaction with the protein are the loops [14,15,16,17]. Moreover, in two recent studies aimed at investigating the T30923/HIV-1 integrase interaction and improving the T30175 biological properties, respectively, we highlighted the relevance of the loops for the aptamer/target interaction and for the integrase-inhibiting ability [18,19]. Besides the already mentioned biological activities, just like other G-rich oligonucleotides, the TBA and T30923 aptamers have also shown antiproliferative/anticancer properties [20,21,22]. Moreover, other aptamers with sequences similar to T30923 have shown interesting biological properties against cancer cell lines. For example, the ODN (Oligodeoxynucleotide) called EAD [T_2_(TG_3_)_4_] has been shown to possess a noteworthy antiproliferative activity against Adriamycin-resistant hormone-dependent breast cancer cell line (MCF-7/ADM) [23], while ODN S13 [(TG_3_)_3_G_3_] has good binding and internalization in lung adenocarcinoma cell line A549 [24]. Recently, a covalent bi-modular version of the sequence [G_3_(TG_3_)_3_] has proven to possess anti-proliferative activity for the neural cancer cell line U87 [25]. Contrary to the significant number of studies concerning the antiproliferative aptamers AS1411 [26,27] (and its analogues [28,29]) and TBA [20] (and its derivatives [30,31,32,33,34,35,36,37,38]), only limited comparable investigations have regarded other aptamers. For example, in the case of the aptamer T30175, no research concerning potential antiproliferative properties has been reported to date, although its sequence partially overlaps those of EAD and S13 and its structure is strictly correlated to that of T30923, as demonstrated by CD (Circular Dichroism), NMR (Nuclear Magnetic Resonance), gel electrophoresis, and molecular dynamics simulations [9,10,39]. As a matter of fact, they both adopt a dimeric head-to-head 5′-5′ end-stacked parallel G-quadruplex structure in which each monomer of the complex is characterized by three G-tetrads and three single-thymidine reversed-chain loops (Figure 1). In particular, differently from T30923, T30175 dimer G-quadruplexes are characterized by an additional bulge loop formed by the extra thymidine in the second position of the sequence (Figure 1) [10]. Hence, in order to evaluate the T30175 antiproliferative potential and the involvement of the residues not belonging to the central core of stacked guanosines in the biological activity, we investigated the structural and biological properties of five T30175 analogues prepared using a single residue replacement approach of sequence thymidines with abasic sites (S) in comparison to the unmodified original aptamer (Table 1).

## 2. Results and Discussion

### 2.1. NMR Spectroscopy

**Figure 2:**
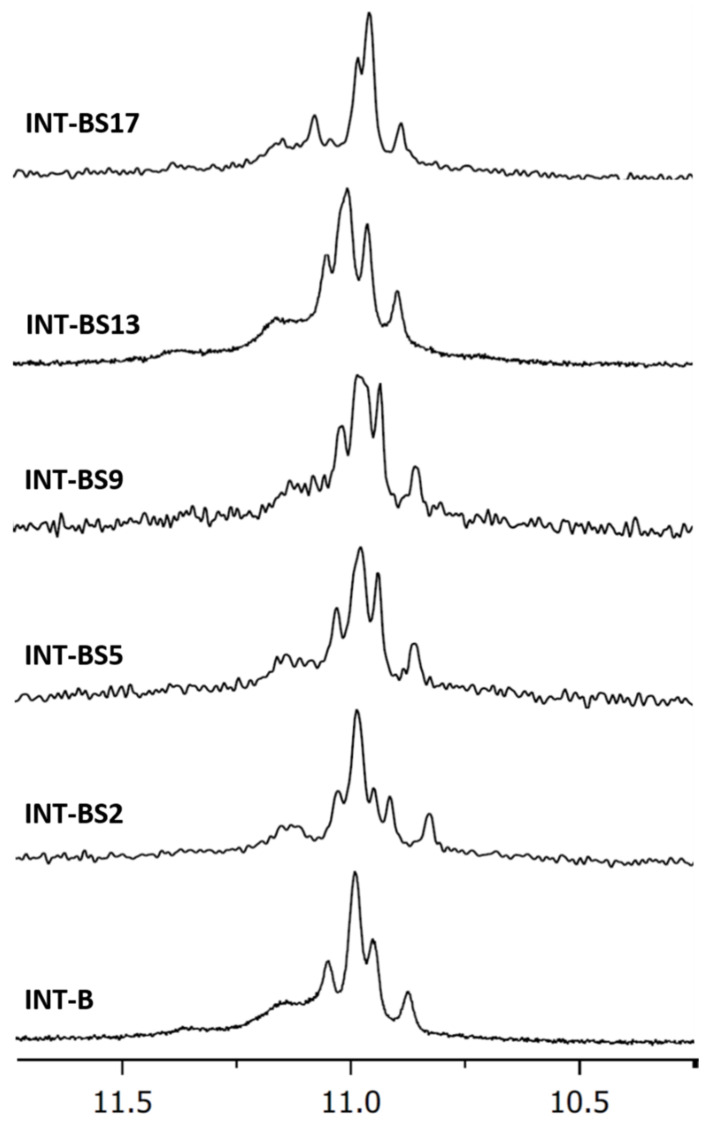
Imino proton regions of the ^1^H-NMR spectra (400 MHz) of T30175 (**INT-B**) and its investigated analogues. See Section 3 for experimental details.

A central issue of this approach is the preliminary verification of the ability of the T30175 derivatives to fold in a parallel G4 structure and to form the characteristic dimeric structure adopted by the unmodified parent sequence. For this reason, ODN analogues were preliminarily investigated by ^1^H-NMR in comparison to their unmodified counterpart (Figure 2) and with the ODN T_2_GTG_2_(TG_3_)_3_T (**TT**-**INT-B**) (Figure S1) consistent with the sequence of T30175, elongated by two additional thymidines at the 5′-end which, in accordance with other authors [10], avoids constitution of the 5′-5′ head-to-head dimer. Figure 2 shows the imino proton regions diagnostic of the presence of G-quadruplex structures (10.5 e 12.0 ppm), in which the strict resemblance between most of the NMR profiles of the T30175 derivatives and their parent dimeric version looks quite clear and the differences from the monomer conformation are rather evident. Slight differences between ^1^H-NMR spectra can be noted for **INT-BS2** and **INT-BS17** [9], as expected because of the peculiar position of S in these derivatives—in the bulge loop and on the 3′-end G tetrad, respectively—thus only negligibly modifying the shape and the position of the imino proton resonances of the G-tetrads. These data clearly indicate that, irrespective of the position in the sequence, a S spacer substituting a thymidine residue does not remarkably impact the dimer conformation assumed by the original structure.

### 2.2. CD Spectroscopy

**Figure 3:**
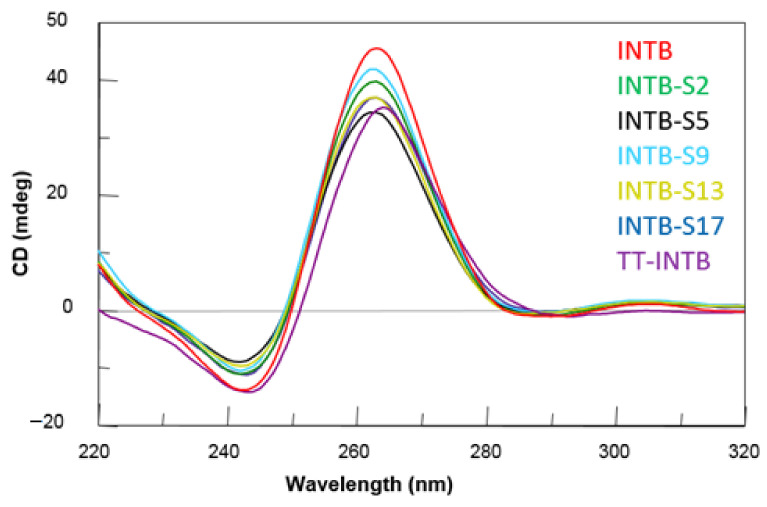
CD spectra of the T30175 analogues investigated. See Section 3 for experimental details.

CD spectra of the modified T30175 aptamers were obtained and compared to their natural counterpart and the ODN T_2_GTG_2_(TG_3_)_3_T (**TT-INT-B**) (Figure 3). All modified sequences exhibited CD profiles almost superimposable on one another and strictly resembling that of the parent aptamer, despite small differences in intensity. These CD profiles revealed a minor negative band at 242 nm and a major positive band at 263 nm, being typical of parallel G-quadruplex structures in which all guanosines assume *anti*-glycosidic conformations. These data confirm NMR results, indicating that our derivatives fold into G-quadruplex conformations firmly, similarly to that of the original aptamer T30175.

With the aim of evaluating the effect of the single replacement of a T residue with an abasic site on the thermal stability of these G-quadruplexes, their melting temperatures (T_m_) were evaluated by CD thermal denaturation experiments. The CD heating profiles of the modified ODNs showed T_m_ values very similar to one another and their natural counterpart (Table 1 and Figure S2), thus demonstrating that the introduction of an abasic site in specific sequence positions does not meaningly impact the conformation and stability of the parent aptamer structure.

### 2.3. Polyacrylamide Gel Electrophoresis (PAGE)

**Figure 4:**
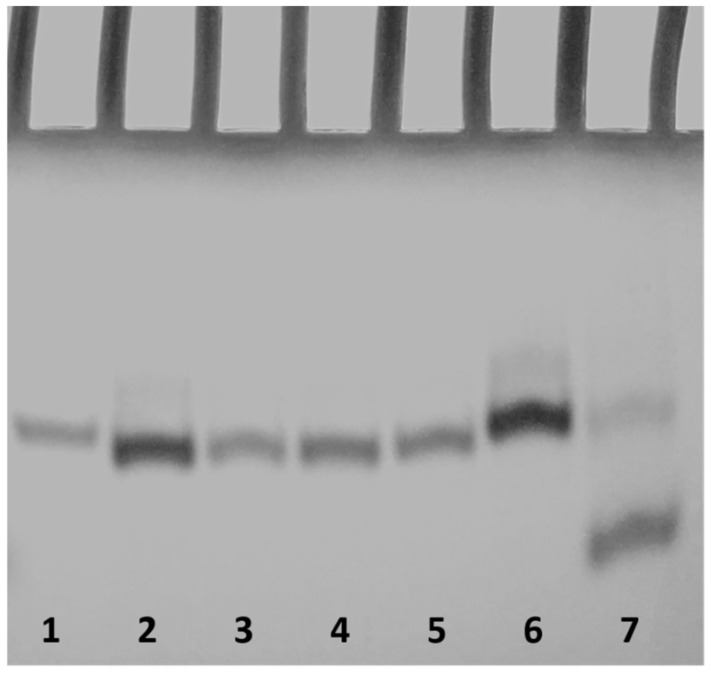
PAGE analysis of the T30175 analogues investigated. Lane 1: **INT-B**; lane 2: **INT-BS2**; lane 3: **INT-BS5**; lane 4: **INT-BS9**; lane 5: **INT-BS13**; lane 6: **INT-BS17**; lane 7: **TT-INT-B**. See Section 3 for experimental details.

In order to afford clear evidence of the presence of dimers for the modified sequences, we examined them by PAGE (Figure 4) in comparison to the natural sequence **INT-B,** which demonstrated the adoption of a 5′-5′ dimer of two stacked parallel G-quadruplexes, and **TT-INT-B,** in which the dimer formation is avoided by the extra thymidines in 5′. The PAGE results unambiguously revealed that **INT-B** and all ODNs containing an abasic site adopt dimeric structures, thus showing slower-migrating bands, while **TT-INT-B** folds mainly into a monomeric G-quadruplex, showing a faster-migrating band. Furthermore, the introduction of abasic sites in the loops slightly affects dimeric species band migration, since these species spot slightly faster-migrating bands than the natural counterpart and **INT-BS17**, in which 3′-dT has been replaced by an abasic site. The PAGE results agree with the data obtained by the other techniques, all indicating that all S derivatives fold into G-quadruplex conformations very similar to the unmodified aptamer.

### 2.4. Nuclease Stability Assay

**Figure 5:**
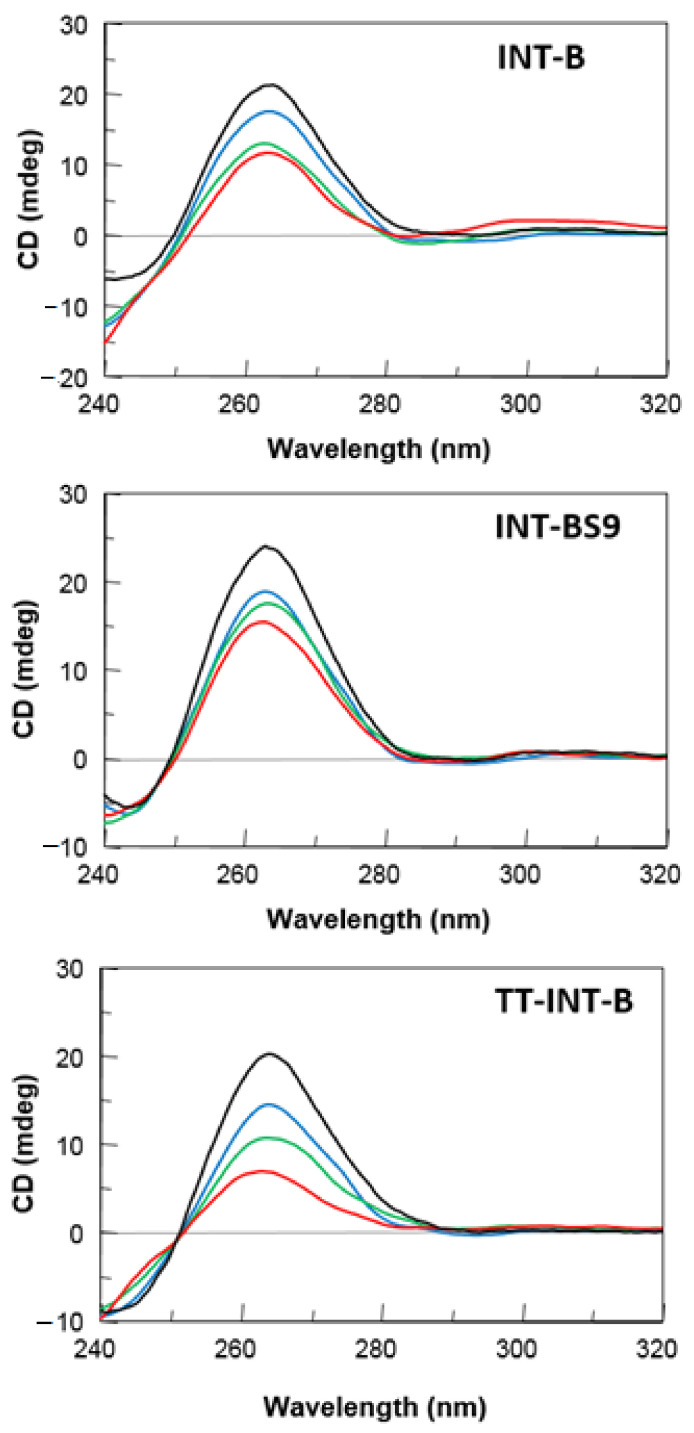
Representative CD spectra of **INT-B**, **INT-BS9**, and **TT-INT-B** in 10% fetal bovine serum (FBS) diluted with Dulbecco’s Modified Eagle’s Medium (DMEM), registered at 0 (black), 24 (blue), 48 (green), and 72 h (red) at 37 °C. See the main text and the Section 3 for details.

To exam the resistance in biological environments, all of the modified ODNs underwent a degradation assay in fetal bovine serum (FBS) and were analyzed by circular dichroism [38] (Figure 5 and Figure S3). To detect typical G-quadruplex CD signals, after subtraction of the background scan (10% FBS in DMEM), the CD spectra of all ODNs were registered in the 220–320 nm region at 0, 24, 48, and 72 h at 37 °C in 10% FBS. All CD profiles showed a time-dependent decrease in band intensities, thus indicating a similar partial resistance to nucleases of both modified and natural G-quadruplex structures, although the decrease in CD signal intensity was more noticeable in the **TT-INT-B** spectrum. Particularly, the CD spectra at 72 h, in the experimental condition used, confirmed the persistence of 55%–65% folded structures for dimeric G-quadruplexes and only 35% for the monomeric one. These data highlight that single T residue replacement with an abasic site in the T30175 aptamer sequence does not affect its resistance to nucleases, and its dimeric species are more resistant than monomeric ones in biological environments.

### 2.5. Antiproliferative Activity

**Figure 6:**
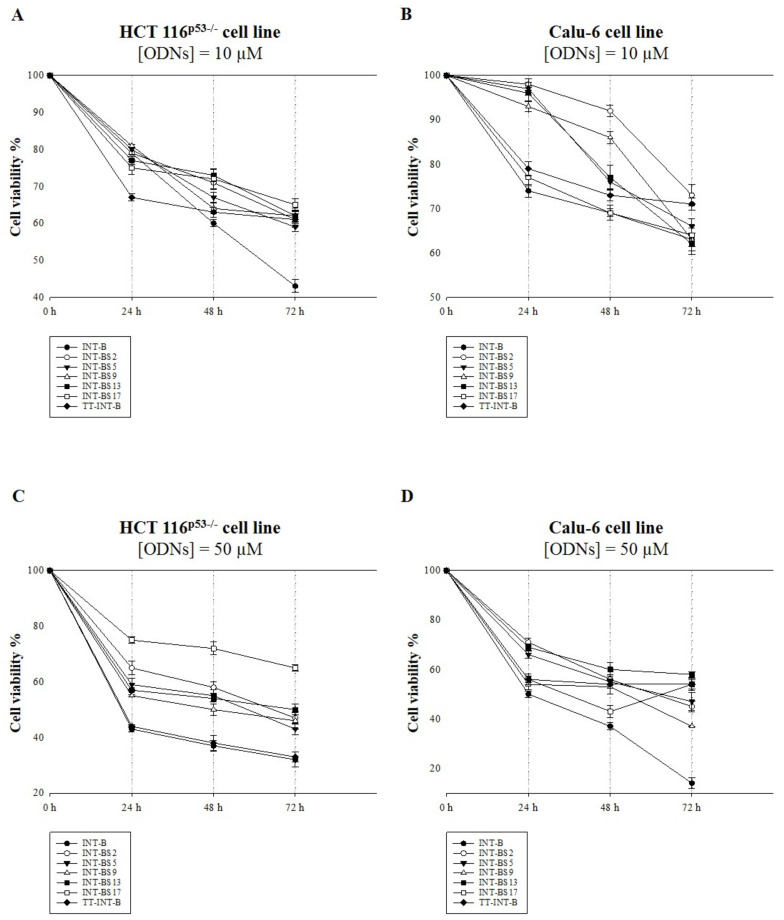
Antiproliferative activity of ODNs on the HCT 116^p53−/−^ (**A**,**C**) and Calu-6 (**B**,**D**) cell lines. Cells were treated with two concentrations of ODNs: 10 μM (**A**,**B**) and 50 μM (**C**,**D**). Cell viability was assayed 24, 48, and 72 h after the addition of ODNs using an MTT assay. The results are presented as a percentage (mean ± SEM) (*n* = 3) of the control cells.

T30175 analogues were evaluated at two different concentrations, 10 and 50 μM, for their antiproliferative activities in Calu-6 and HCT 116^p53−/−^ cells, i.e., lung and colon cancer cell lines, respectively, in comparison to their natural counterpart in dimeric and monomeric forms. For this purpose, cells were incubated with ODNs for 24, 48, and 72 h. Then, cell viability was examined using an MTT assay. In both the Calu-6 and HCT 116^p53−/−^ cell lines, all T30175 analogues showed an antiproliferative effect (Figure 6). The most marked effect on cell growth was found in the HCT 116^p53−/−^ cell line by **INT-B** at both doses and in a time-dependent manner. In particularly, we observed that **INT-B** reduced colon cancer cell viability by approximately 50% and 70% at 10 and 50 μM, respectively, after 72 h of treatment. The other ODNs were less effective at inhibiting cell viability in these cancer cells (Figure 6A–C). Also in Calu-6 cells, **INT-B** showed the greatest antiproliferative activity at both concentrations. Remarkably, at 50 μM and after 72 h of treatment, **INT-B** resulted in a reduction of 90% of cell viability (Figure 6B–D). Recently, some authors have highlighted the influence of G-quadruplex structural elements on the antiproliferative properties of certain G-rich oligonucleotides [40]. In our case, it is interesting to note that in both cell lines, the most significant antiproliferative results were related to **INT-B**, the unique ODN characterized by an intact dimeric G-quadruplex structure, suggesting that both its typical folding in dimeric form and the presence of all thymidines in the sequence are pivotal features for this biological activity.

### 2.6. Anti-Motility Property

Starting from these results and to better characterize the potential anticancer activity of all derivatives, we investigated their effects on Calu-6 cell motility by a wound healing assay. We also tested **TT-INT-B**, since it is the only modified ODN that adopts a monomeric G-quadruplex structure. For this purpose, cells were treated with 25 μM of **each ODN** and quantitatively evaluated in terms of percentage of the open wound after 12 and 24 h of treatment in comparison to untreated cells.

**Figure 7:**
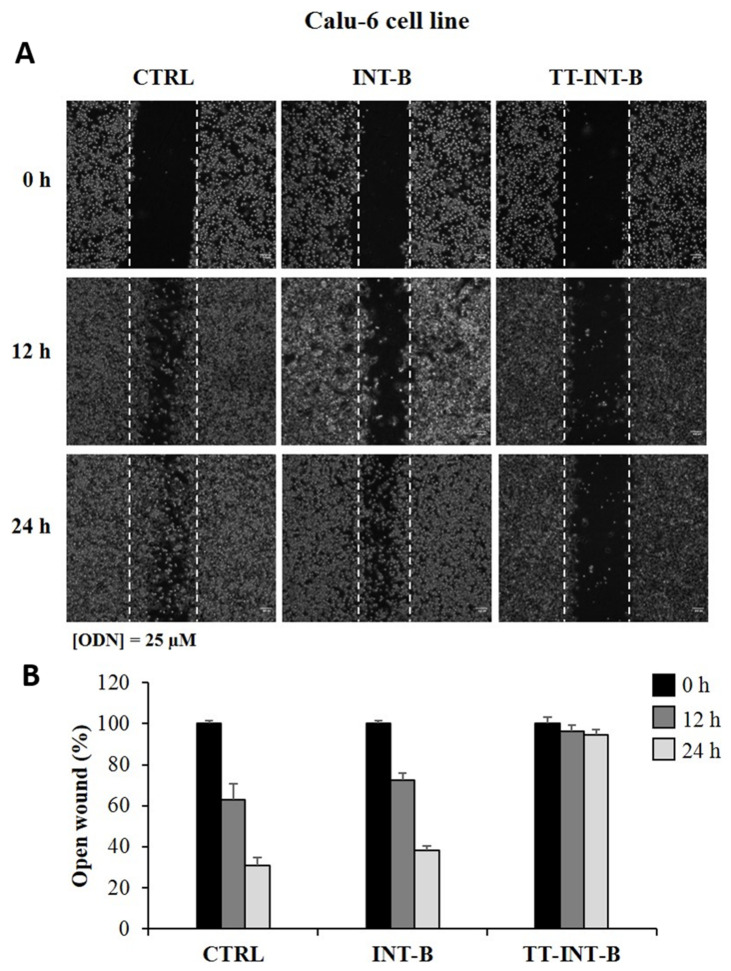
Effects of **INT-B** and **TT-INT-B** on cell migration. Representative images of the wound healing assay in the Calu-6 cell line (**A**). The cells were treated with 25 µM of **INT-B** and **TT-INT-B** for 24 h. Wound widths were measured at 0, 12, and 24 h on three fields per well and averaged. Data are expressed as the fold-decrease of area with respect to the control set as 100% (**B**). Bars represent the mean of triplicate experiments; error bars represent the standard deviation.

Our results showed that the wound healing ability of cells treated with **INT-B** decreased only slightly compared to that observed in the control, namely, untreated cells. In particular, after 24 h of **INT-B** treatment, cells filled approximately 60% of the wound area in comparison to 70% obtained in control cells at the same time (Figure 7A,B). Interestingly, all T30175 analogues containing a S spacer exhibited anti-motility activities to some extent (Figure S4).

Otherwise, treatment with **INT-BS9** and **TT-INT-B** completely abolished the wound healing ability of these cells. Indeed, at 24 h, the wound was completely unclosed, while, at the same time, the control cells had filled approximately the 70% of the wound area. These data suggest that both the T30175 monomeric form and the dimeric modified aptamers can be involved in counteracting the migratory activity of Calu-6 cell line.

A comparison between the results obtained in the MTT and wound healing assays showed that the antiproliferative and anti-migratory activities of T30175 and its derivatives depend on different structural features able to activate distinct specific molecular pathways.

## 3. Materials and Methods

### 3.1. Oligonucleotide Synthesis and Purification

The modified ODNs listed in Table 1 were synthesized by an ABI 394 DNA synthesizer using solid-phase β-cyanoethyl phosphoramidite chemistry at the 10 µmol scale. The synthesis was carried out using normal 3′-phosphoramidites and a 5′-dimethoxytrityl-3′-phosphoramidite-1′,2′-dideoxyribose (dSpacer, S, Link Technologies, Glasgow, UK) for the introduction of an abasic site mimic moiety in each sequence. For ODN **INT-BS17**, a universal support was used. The detachment from the support and the deprotection of the oligomers were carried out by treatment with concentrated aqueous ammonia at 80 °C overnight. The combined filtrates and washings were concentrated under reduced pressure, redissolved in H_2_O, analyzed, and purified by high-performance liquid chromatography on a Nucleogel SAX column (Macherey-Nagel, Duren, Germany, 1000-8/46) using buffer A (20 mM NaH_2_PO_4_/Na_2_HPO_4_ aqueous solution (pH 7.0) containing 20% (*v*/*v*) CH_3_CN) and buffer B (1 M NaCl, 20 mM NaH_2_PO_4_/Na_2_HPO_4_ aqueous solution (pH 7.0) containing 20% (*v*/*v*) CH_3_CN); a linear gradient from 0% to 100% B for 45 min and a flow rate of 1 mL/min were used. The fractions of the oligomers were collected and successively desalted by Sep-pak cartridges (C-18). The isolated oligomers proved to be >98% pure by NMR.

### 3.2. NMR Spectroscopy

NMR samples were prepared at a concentration of approximately 2 mM in 0.6 mL (H_2_O/D_2_O 9:1 *v*/*v*) of buffer solution with 10 mM KH_2_PO_4_/K_2_HPO_4_, 70 mM KCl, and 0.2 mM EDTA (pH 7.0). All of the samples were heated for 5–10 min at 90 °C and slowly cooled (10–12 h) to room temperature. The solutions were equilibrated for several hours at 4 °C. The annealing process was assumed to be complete when the ^1^H NMR spectra were superimposable on changing time. NMR spectra were recorded with a Varian Unity INOVA 500 MHz spectrometer. 1D proton spectra of the samples in H_2_O were recorded using pulsed-field gradient DPFGSE for H_2_O suppression [41]. ^1^H-chemical shifts were referenced relative to external sodium 2,2-dimethyl-2-silapentane-5-sulfonate (DSS).

### 3.3. CD Spectroscopy

CD samples of **INT-B** and its derivatives were prepared at an ODN concentration of 50 µM using a potassium phosphate buffer (10 mM KH_2_PO_4_/K_2_HPO_4_, 70 mM KCl, pH 7.0) and submitted to the annealing procedure (heating at 90 °C and slowly cooling at room temperature). The CD spectra of all quadruplexes and the CD melting curves were registered on a Jasco 715 CD spectrophotometer by taking the average of three scans. For the CD spectra, the wavelength varied from 220 to 320 nm at a 100 nm min^−1^ scan rate, and the spectra were recorded with a response of 16 s at a 2.0 nm bandwidth and normalized by subtraction of the background scan with the buffer. The temperature was kept constant at 20 °C with a thermoelectrically controlled cell holder (Jasco PTC-348). The CD melting curves were registered as a function of temperature (range: 20–90 °C) for all quadruplexes at their maximum Cotton effect wavelengths. The CD data were recorded in a 0.1 cm pat–length cuvette with a scan rate of 10 °C/h. The melting temperature (Tm) values provided the best fit of the experimental melting data.

### 3.4. Gel Electrophoresis

All oligonucleotides were tested by non-denaturing PAGE. Samples in the CD buffer (10 mM KH_2_PO_4_/K_2_HPO_4_, 70 mM KCl, pH 7.0) were loaded on a 20% polyacrylamide gel containing Tris–Borate-EDTA (TBE) 2.5× and KCl 20 mM. The run buffer was TBE 1× containing 50 mM KCl. For all samples, a solution of glycerol/TBE 10× was added just before loading. Electrophoresis was registered at 8 V/cm at a temperature close to 10 °C. The bands were visualized by UV shadowing.

### 3.5. Nuclease Stability Assay

A nuclease stability assay of the modified ODNs was conducted in 10% fetal bovine serum (FBS) diluted with Dulbecco’s Modified Eagle’s Medium (DMEM) at 37 °C and studied by CD analysis. Approximately 7 nmol of stock solution of each ODN (~1 O.D.U.) was evaporated to dryness under reduced pressure and then incubated with 250 μL of 10% FBS at 37 °C. The degradation patterns were analyzed by monitoring the CD signal decrease of each sample at 37 °C, as a function of time. The CD spectra at 0, 24, 48, and 72 h for all ODNs were recorded at 37 °C using a Jasco 715 spectrophotometer equipped with a Peltier temperature control system (Jasco, Tokyo, Japan). Data were collected from 240 to 320 nm with a 1 s response time and a 1 nm bandwidth using a 0.1 cm quartz cuvette. Each spectrum shown was corrected for the spectrum of the reaction medium (10% FBS in DMEM).

### 3.6. Cell Cultures and Treatments with the ODNs

The Calu-6 and HCT 116^p53−/−^ cell lines were cultured as antecedently described [37]. Treatment of cells was achieved by substituting the culture medium with those containing ODNs at final concentrations of 10, 25, and 50 μM.

### 3.7. MTT Assay

The Calu-6 [42] and HCT 116^p53−/−^ cells [43] were seeded onto 96-well plates at the proper density and treated with different ODNs at final concentrations of and 50 μM from 24 to 72 h. Then, cell viability was analyzed via an MTT assay as antecedently described [32]. A pool of three different sets of experiments, each repeated in triplicate, were performed. Error bars represent the mean ± standard deviation (SD) from *n* = 3 biological replicates. Statistical comparisons were conducted as previously shown [43].

### 3.8. Wound Healing Assay

Cell motility was assessed using a wound healing assay as previously reported [44]. Briefly, Calu-6 cells (1 × 10^6^ per well) were seeded into 35 mm tissue culture plates. The confluent monolayer cells were carefully wounded using a sterilized pipette tip. Then, the cells were treated with ODNs at a final concentration of 25 μM. Monolayer cells were photographed at 0, 12, and 24 h with an objective of 10×. Quantitative analysis of the wound assay was carried out by measuring the gap area. The gap area was defined using ImageJ software (National Institute of Health, Bethesda, MD, USA (US-MD)). Data are expressed as the fold-decrease of area with respect to the control set as 100%. Bars represent the mean of triplicate experiments; error bars represent the standard deviation.

## 4. Conclusions

In this investigation, the structural and biological properties of five T30175 derivatives containing an abasic site (S) singly replacing the thymidines of loops were examined in comparison to their natural counterpart. The design of these analogues was founded on the plainness that G-quadruplex aptamers could be especially appropriate to be analyzed by this strategy, because it is rightful to think that most of the structural stability is based on a scaffold of stacked G-tetrads, while the loop residues, being projected externally, are more likely involved in the interaction with the target and, then, prone to be substituted by an abasic site in order to study in depth their role and function in the structure/activity relationship. The synthesis of the derivatives containing S is easy and founded on standard procedures, and the collected NMR, CD, and PAGE data strongly suggest that all of them adopt G4 structures strictly similar to those of the parent aptamer, with the ability to fold into a dimeric structure formed by the same two G-quadruplexes, each characterized by parallel strands, three all-*anti* G-tetrads and four one-thymidine loops (one bulge and three propeller loops). The data concerning the nuclease stability assay showed that single T residue replacement with an abasic site in a T30175 aptamer sequence does not significantly affect its resistance to nucleases, and that its dimeric species are more resistant than that of monomeric ones in biological environments. However, though all ODNs investigated here exhibited anti-proliferative activity against Calu-6 lung and HCT 116^p53−/−^ colorectal cancer cells, the unmodified T30175 aptamer showed the greatest effect on cell growth at both doses tested (10 and 50 μM), in a time-dependent manner in HCT 116^p53^^−/−^ cell line. Additionally, also in Calu-6 cells, the parent aptamer displayed the greatest antiproliferative activity at both concentrations, with the remarkable result of a 90% decrease in cell viability at 50 μM after 72 h of treatment. These data represent the first evidence of the antiproliferative ability of T30175 in different cancer cells, thus revealing that the G-quadruplex inhibitory activity is strongly dependent on its structure integrity, suggesting that both the typical folding in dimeric form and the presence in the sequence of all thymidines are crucial features for this biological activity. Moreover, an unprecedented anti-motility ability was observed for the monomeric form, **TT-INT-B**, and the dimeric modified aptamers, particularly **INT-BS9**. In fact, unlike the results obtained from the MTT assay, the wound healing assay on Calu-6 cells highlighted that the wound healing ability of these cells is completely abolished by treatment with **TT-INT-B** and **INT-BS9**, while only partially by treatment with other modified dimeric quadruplexes. Otherwise, the migratory property of cells handled with the unmodified dimeric form **INT-B** was only slightly reduced in comparison to that observed in the untreated sample. These results are not particularly surprising, taking into consideration that the antiproliferative and anti-migratory activities are associated with different, specific, and multifactorial molecular pathways, probably activated by different structural features of the investigated ODNs. Considering the possibility to introduce different types of modified residues into specific positions of loops and the availability of modified thymidine phosphoramidites, obtained by commercial sources or prepared by synthetic approaches antecedently reported, our inspiring results corroborate the use of this straightforward approach to understand the critical requirements of the G-quadruplex structures that affect the antiproliferative potential and suggest its application to other G-quadruplex aptamers with structural features comparable to T30175.

## Figures and Tables

**Table 1:** Sequence and melting temperature (Tm) of the investigated ODNs. S indicates an abasic site mimic.

Oligonucleotide	Sequence	T_m_ (°C) ± 1
INT-B (T30175)	5′-GTGGTGGGTGGGTGGGT-3′	86
INT-BS2	5′-GSGGTGGGTGGGTGGGT-3′	86
INT-BS5	5′-GTGGSGGGTGGGTGGGT-3′	84
INT-BS9	5′-GTGGTGGGSGGGTGGGT-3′	85
INT-BS13	5′-GTGGTGGGTGGGSGGGT-3′	84
INT-BS17	5′-GTGGTGGGTGGGTGGGS-3′	83
TT-INT-B	5′-TTGTGGTGGGTGGGTGGGT-3′	84

## Data Availability

Data is contained within the article or Supplementary Materials.

## Supplementary Materials

The following are available online at https://www.mdpi.com/article/10.3390/ijms22137040/s1.
