# Supplementary material for: Exploring New Potential Anticancer Activities of the G-Quadruplexes Formed by [(GTG2T(G3T)3] and Its Derivatives with an Abasic Site Replacing Single Thymidine
Source: Int J Mol Sci. 2021 Jun 30;22(13):7040. doi: 10.3390/ijms22137040 (PMC8268168; doi:10.3390/ijms22137040)
Supplement: Supplementary file 1 [file ijms-22-07040-s001.zip › ijms-1264179-supplementary.pdf]

Supplementary Material

Exploring new potential anticancer activities of the G-quadruplexes formed by [(GTG$_2$T(G$_3$T)$_3$] and its derivatives with an abasic-site replacing single thymidine.

Antonella Virgilio[a], Daniela Benigno[a], Annalisa Pecoraro[a], Annapina Russo[a], Giulia Russo[a], Veronica Esposito[a]* and Aldo Galeone[a].

[a] Department of Pharmacy, University of Naples Federico II, Napoli, Italy.

* Corresponding author: VE: verespos@unina.it.

Table of contents

1H-NMR spectrum

CD melting profiles

CD spectra in 10% Fetal Bovine Serum (FBS)

Wound Healing Assay

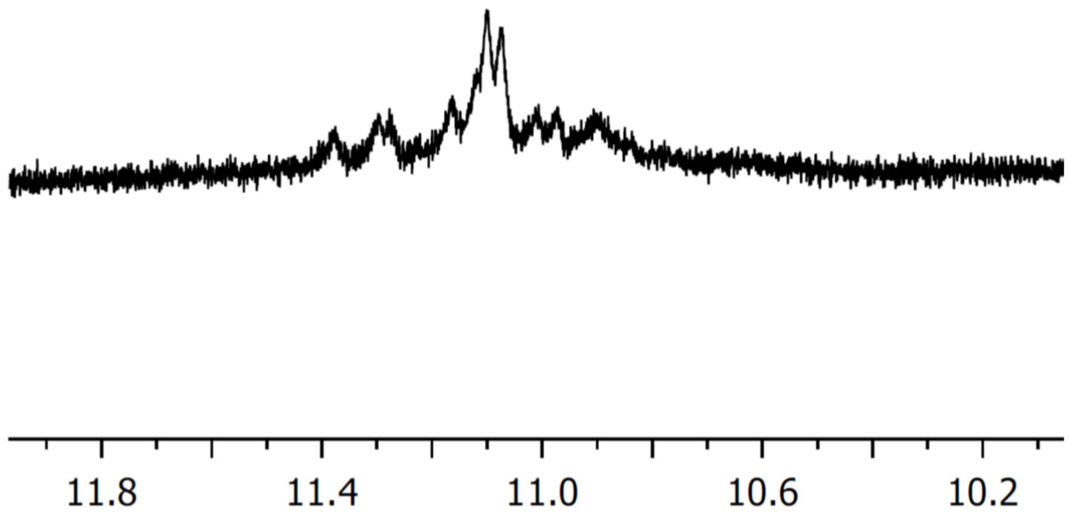

Figure S1. Imino proton region of the ^{1}H-NMR spectrum (400 MHz) of **TT-INT-B.** See Materials and Methods for experimental details.

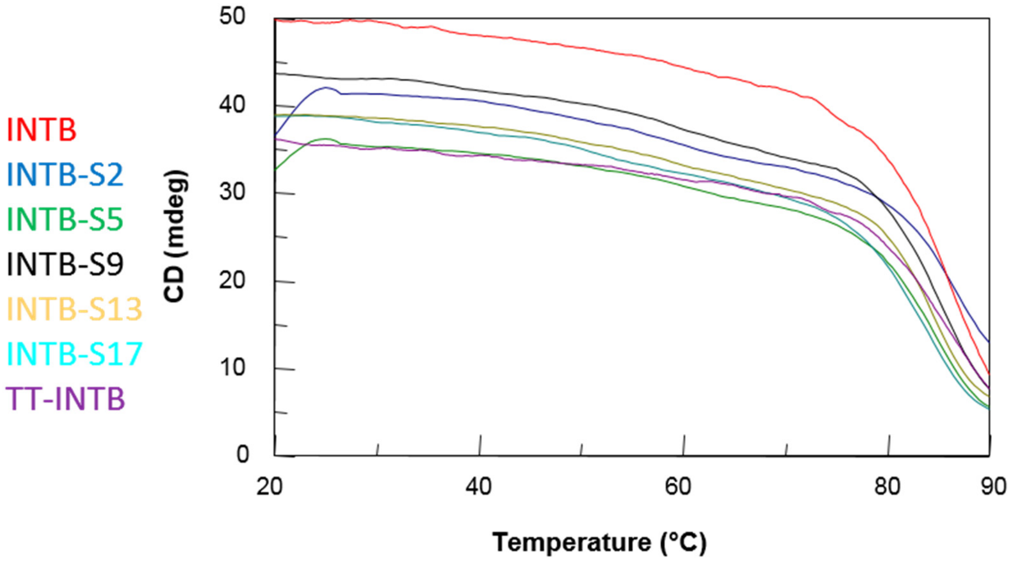

Figure S2. CD melting profiles of analyzed ODNs registered as a function of temperature for all modified quadruplexes at their maximum Cotton effect wavelengths. CD data were recorded in a 0.1 cm pathlength cuvette with a scan rate of 10°C/h at 50 µM ODN strand concentration in potassium phosphate buffer (10 mM KH_2PO_4/K_2HPO_4, 70 mM KCl, pH 7.0).

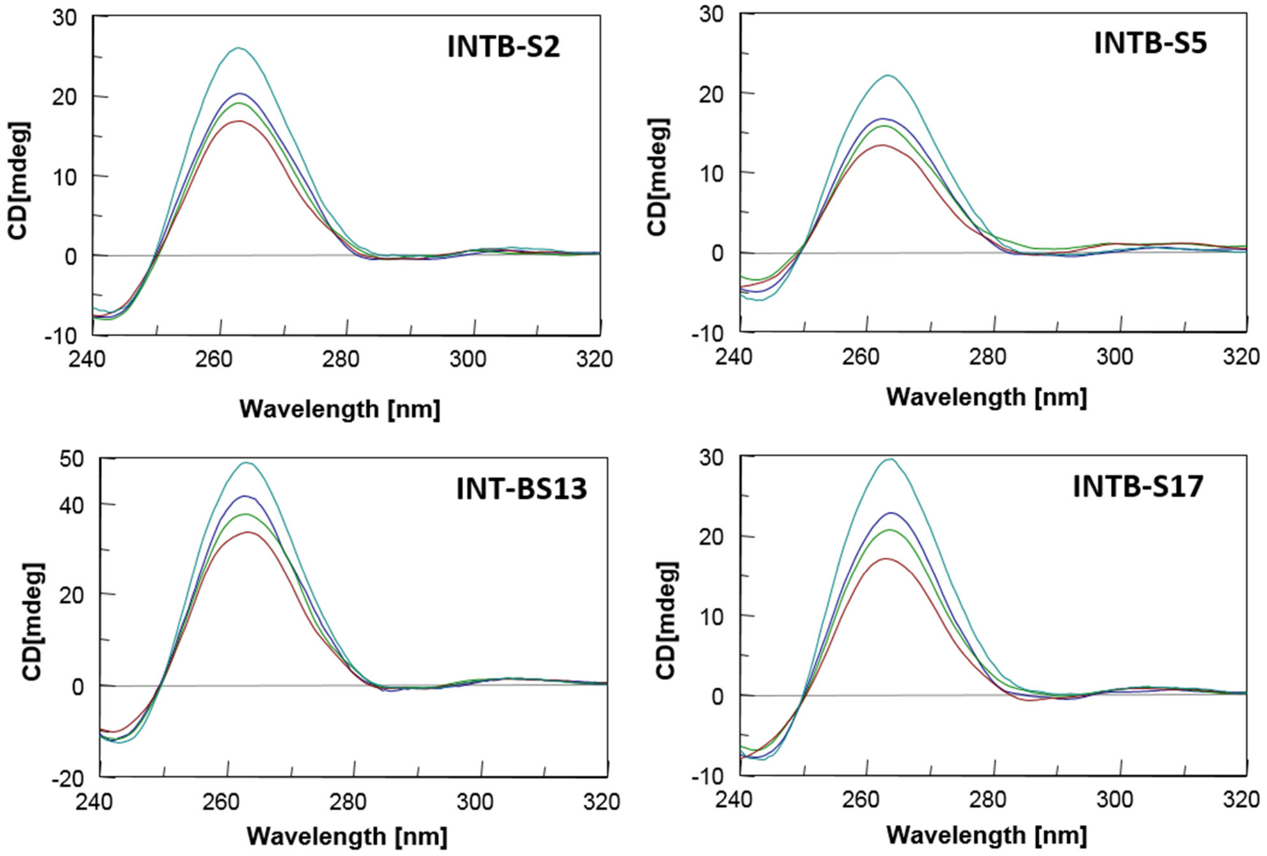

Figure S3. CD spectra of **INT-BS2**, **INT-BS5**, **INT-BS13** and **INT-BS17** in 10% Fetal Bovine Serum (FBS) diluted with Dulbecco's Modified Eagle's Medium (DMEM), registered at 0 (light blue), 24 (blue), 48 (green) and 72 h (red), at 37°C. See the main text and the Materials and Methods section for details.

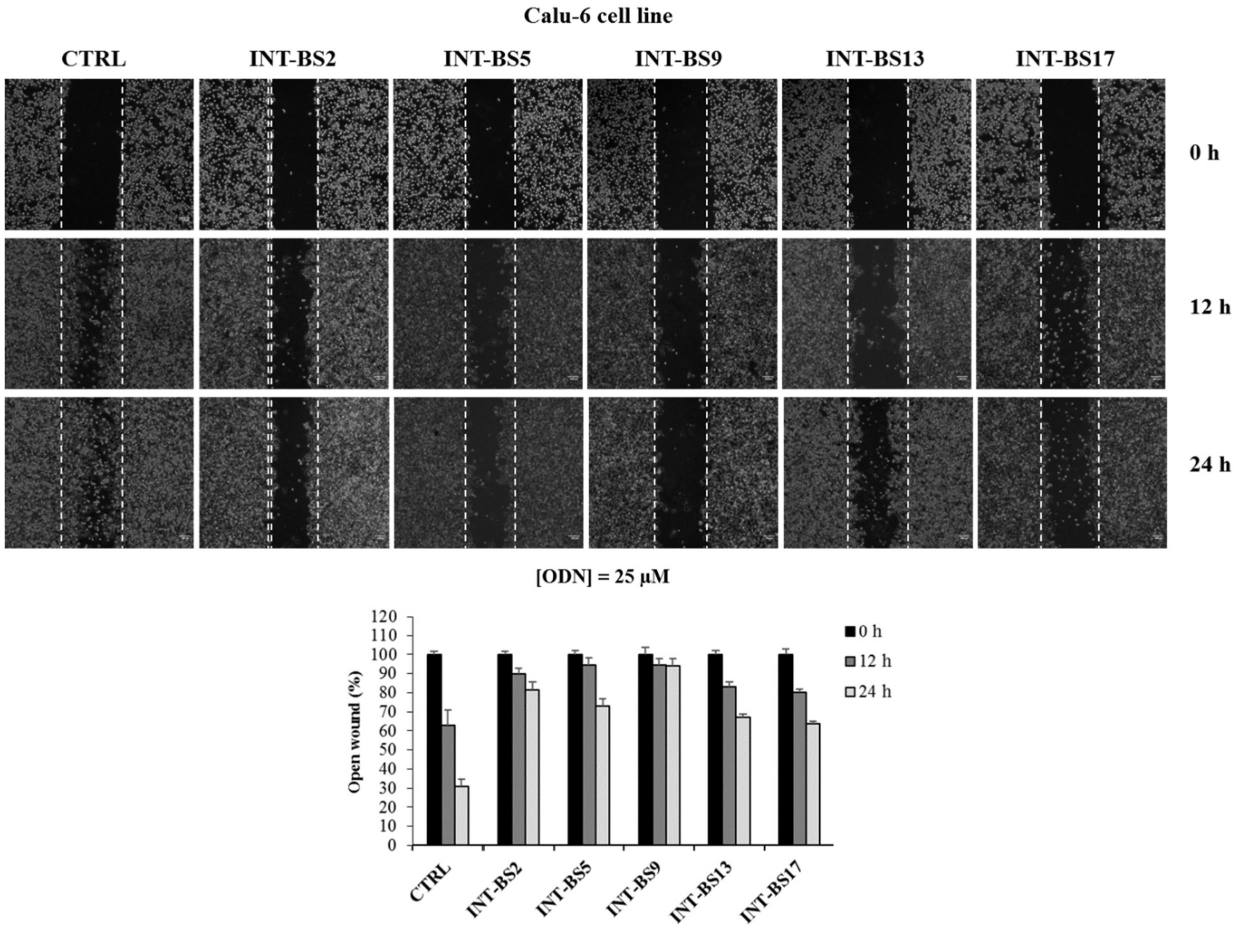

Figure S4. Effects of INT-B derivatives on cell migration. Representative images of wound healing assay in Calu-6 cell line (on the top). Cells have been treated with 25 µM of ODNs for 24 h. Wound widths were measured at 0, 12 and 24 h on 3 fields per well and averaged. Data have been expressed as the fold-decrease of area respect to control set as 100% (on the bottom). Bars represent the mean of triplicate experiments; error bars represent the standard deviation.
